# A Rare Pathogenic Masquerader of Pulmonary Disease: A Case Report

**DOI:** 10.7759/cureus.78822

**Published:** 2025-02-10

**Authors:** Rayhan Karimi, Arun Adlakha

**Affiliations:** 1 Internal Medicine, Edward Via College of Osteopathic Medicine, Spartanburg, USA; 2 Pulmonology, Carolina Lung Clinic, Piedmont Medical Center, Rock Hill, USA

**Keywords:** acid-fast bacteria, copd, mycobacterium szulgai, ripe therapy, upper lobe cavitations

## Abstract

This case report details a 61-year-old male patient with a history of chronic obstructive pulmonary disease (COPD) and significant smoking exposure, who presented with progressive anorexia, weight loss, nocturnal sweating, and worsening respiratory symptoms in early 2024. Imaging revealed bi-apical opacities with cavitation, and sputum cultures identified *Mycobacterium szulgai*, a rare nontuberculous mycobacterium (NTM). The patient was managed with a multi-drug regimen including azithromycin, rifampin, and ethambutol, targeting symptom relief, radiographic stability, and sputum conversion. Early treatment results demonstrated clinical improvement, weight gain, and decreased radiographic abnormalities, although sputum studies remained positive. This case underscores the diagnostic and therapeutic complexities of rare NTM infections in patients with underlying lung disease.

## Introduction

Nontuberculous mycobacteria (NTM) are opportunistic pathogens that can cause a range of infections, particularly in individuals with underlying structural lung diseases such as chronic obstructive pulmonary disease (COPD), bronchiectasis, or prior pulmonary tuberculosis [1]. Additional risk factors include immunodeficiency and immunosuppression. Among the numerous NTM species, *Mycobacterium szulgai *is one of the rarest, accounting for less than 0.01% of all NTM infections [1]. It has been predominantly associated with pulmonary involvement, although it can occasionally present with extrapulmonary manifestations.

The clinical presentation of *M. szulgai* pulmonary disease often mimics more common infections such as tuberculosis or other NTM infections, making diagnosis challenging. The infection is characterized by non-specific symptoms such as chronic cough, fatigue, weight loss, and dyspnea, along with radiographic findings that can include nodules, cavitation, or consolidation, often in the upper lung zones. Of the 11,039 cases of NTM reported over the time period of 1995-2006 in the UK, *M. szulgai* was identified as a causative organism in only 30 cases [2]. Given its rarity, there is limited data in the existing literature regarding the diagnosis, investigation, and long-term outcomes of *M. szulgai* infections.

This report describes a case of *M. szulgai* pulmonary disease in a 61-year-old male patient with COPD, highlighting the diagnostic challenges, therapeutic considerations, and clinical course. The report aims to contribute to the growing body of literature on this uncommon pathogen and emphasize the importance of early identification and appropriate treatment in achieving favorable outcomes.

## Case presentation

A 61-year-old male patient with a past medical history of anxiety, depression, hyperlipidemia, osteoarthritis, acid reflux, benign liver and adrenal cysts, and a 35+ pack-year smoking history was evaluated in the fall of 2021 for the presence and severity of underlying chronic obstructive pulmonary disease. He presented with chronic intermittent mucoid productive cough, wheezing, shortness of breath, and fatigue. He denied fever, anorexia, weight loss, sweating, hemoptysis, or chest pain. His family history was significant for a mother with rheumatoid arthritis and myocardial infarction, and a father with depressive disorder. Surgical history included an appendectomy. Socially, he worked as a truck driver and reported exposure to burning wood, plastics, and construction environments. Medication history included daily use of albuterol hydrofluoroalkane (HFA), omeprazole, rosuvastatin, buspirone, fluoxetine, cyclobenzaprine, baclofen, diclofenac, and tizanidine.

On general examination, he appeared well-nourished and in no apparent distress. His vital signs were normal, and his resting room air oxygen saturation was 99%. His body mass index (BMI) was 24 kg/m². Chest examination revealed few expiratory rhonchi in both lower lungs and a diffuse prolonged expiratory phase of respiration. The remainder of the systemic examination was unremarkable. Baseline office spirometry demonstrated moderate obstructive airway disease with a forced expiratory volume in one second (FEV₁)/forced vital capacity (FVC) ratio of 0.65 and a FEV₁ of 68% predicted (Table 1).

**Table 1:** Patient laboratory values with comparison to reference ranges BMI: body mass index, FEV₁: forced expiratory volume in one second, FVC: forced vital capacity

Parameters	Patient values	Reference values
BMI	24 kg/m^2^	18.5-24.9 kg/m^2^
Oxygen saturation	99%	>90%
FEV_1_/FVC ratio	0.65	0.7-0.8
FEV_1_	68%	80%-120%

**Figure 1:**
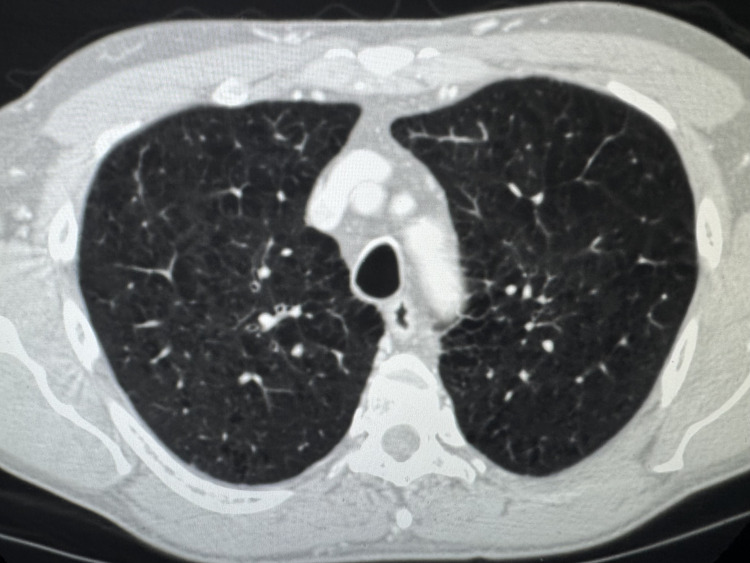
Axial section of low-dose chest CT showing bilateral, upper, and middle lung centrilobular emphysema CT: computed tomography

Low-dose computed tomography (CT) of the chest revealed bilateral upper lung centrilobular emphysema without evidence of cavitation, nodules, consolidation, atelectasis, bronchiectasis, pleural disease, mediastinal adenopathy, or other significant abnormalities (Figure 1).

He was initiated on a triple combination of an inhaled long-acting muscarinic antagonist, a long-acting beta-agonist, and an inhaled corticosteroid. He was advised to continue the as-needed use of an inhaled short-acting beta-agonist for symptomatic relief.

Over the following years, his COPD symptoms remained stable. Spirometry revealed FEV₁ oscillating between 65% and 72% predicted, and he progressively reduced daily cigarette use. He did not experience COPD exacerbations requiring antibiotics or systemic steroids. His other medical conditions also remained stable, and he did not develop any new disorders or undergo additional surgical procedures.

**Figure 2:**
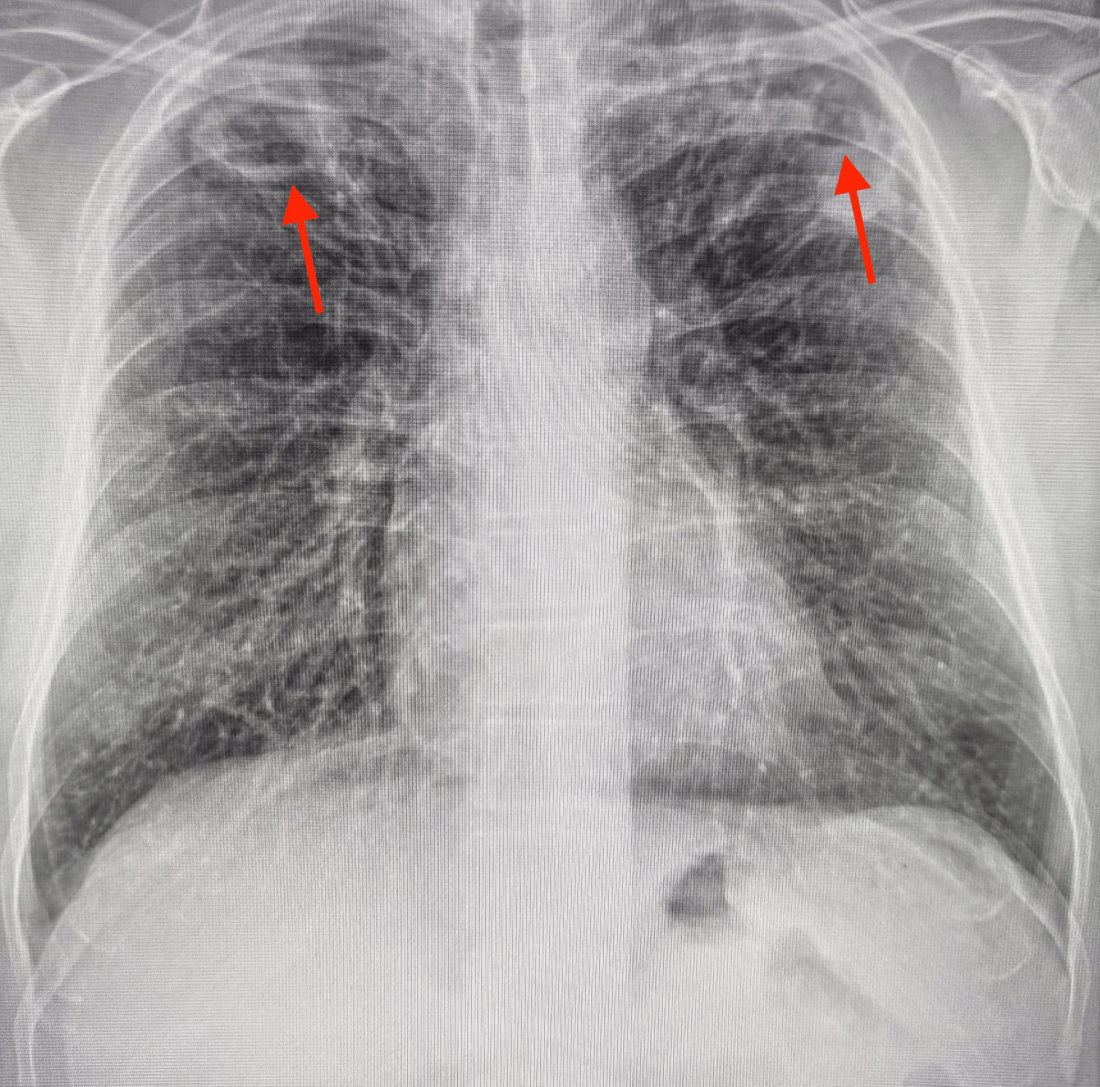
Anterior-posterior chest X-ray showing bi-apical opacities with cystic lucencies (arrows)

In early 2024, he reported a subacute onset of anorexia, weight loss, nocturnal sweating, increased baseline fatigue, dyspnea on exertion, mucoid productive cough, and reduced energy levels. He denied recent travel, exposure to bat or bird droppings, tuberculosis, or healthcare-related risks. On examination, his BMI had declined to 22 kg/m². Resting room air oxygen saturation remained stable at 99%. Systemic examination was inconclusive. Chest X-ray showed bi-apical opacities with cystic lucencies (Figure 2).

**Figure 3:**
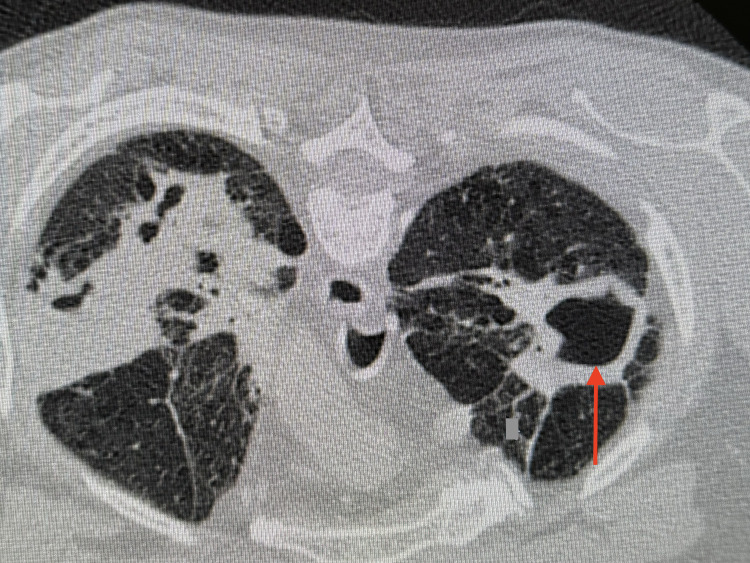
Axial sections of chest CT with contrast that show large areas of cavitation within the upper lobes of the lungs (arrow) CT: computed tomography

**Figure 4:**
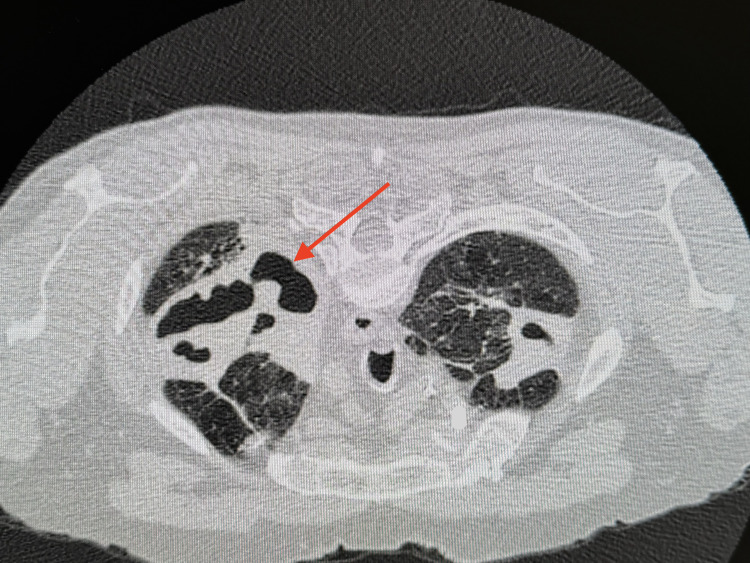
Axial sections of chest CT with contrast that show large areas of cavitation within the upper lobes of the lungs (arrow) CT: computed tomography

CT imaging revealed large areas of cavitation within the upper lobes of the lungs, more pronounced on the left than the right (Figures 3, 4). Mildly prominent but non-pathologically enlarged mediastinal lymph nodes were noted.

Laboratory findings included a normal complete blood count (CBC) and comprehensive metabolic panel. Fungal beta-D glucan assay (Fungitell) and Aspergillus IgG were negative, and the QuantiFERON-TB Gold Plus test was negative on two occasions. Early morning sputum studies for bacterial, fungal, and acid-fast bacilli (AFB) smears and cultures were obtained. Regular sputum cultures grew normal respiratory flora, and no fungi were isolated. Polymerase chain reaction (PCR) testing for *Mycobacterium tuberculosis *was negative. Sputum cultures eventually grew *Mycobacterium szulgai*, taking about six weeks from tissue sampling to identification. Susceptibility testing showed sensitivity to clarithromycin, rifampin, amikacin, moxifloxacin, and rifabutin (Table 2).

**Table 2:** Susceptibility testing results AFB: acid-fast bacilli

Antibiotic	Susceptibility (µg/mL or ratio)
Trimeth/Sulfa	>4/76
Amikacin	1
Ciprofloxacin	8
Clarithromycin	0.5
Doxycycline	4
Linezolid	4
Minocycline	2
Moxifloxacin	1
Rifabutin	1
Rifampin	2
Streptomycin	4

Infectious disease consultation was sought. Blood and urine Histoplasma galactomannan antigen tests were negative. The patient declined a CT-guided or bronchoscopy-guided tissue biopsy. He agreed to initiate therapy with azithromycin 500 mg daily, rifabutin 600 mg daily, and ethambutol 800 mg daily. Treatment goals included improvement of symptoms, prevention of radiographic progression, preservation of pulmonary function, and conversion of sputum. An intensive phase of at least three drugs for three months was advised. The expected duration of therapy was 12 months after achieving consistently negative sputum cultures for *M. szulgai*.

At his most recent follow-up, the patient reported clinical improvement, including reduced productive sputum, less fatigue, and better breathing. He had reduced cigarette use to only a few per day and tolerated anti-tuberculosis medications without major side effects. An ophthalmology evaluation, required due to ethambutol treatment, was normal. He had gained 4 pounds, and his general and systemic examinations were unremarkable.

**Figure 5:**
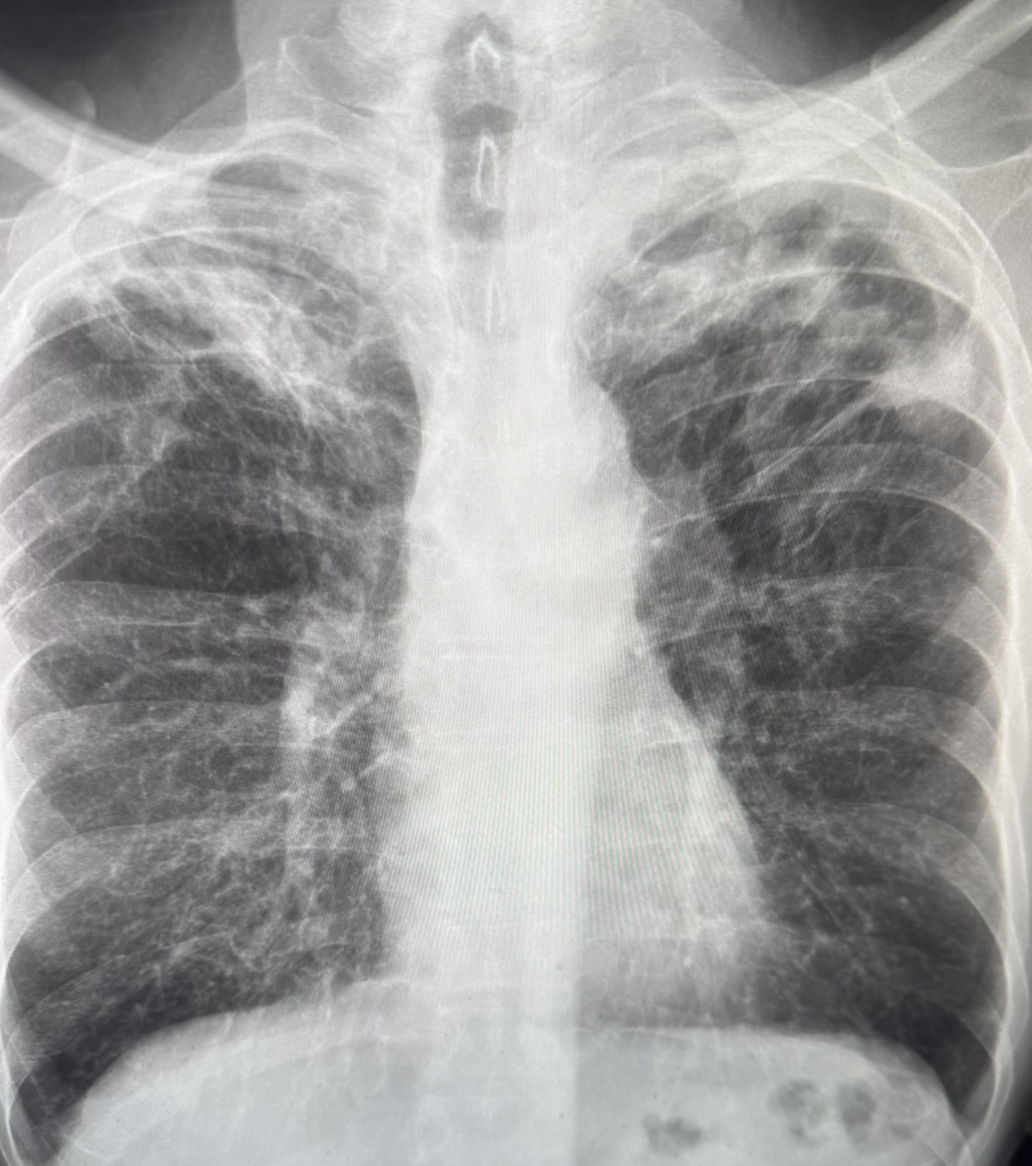
Anterior-posterior chest X-Ray revealing bi-apical lung fibrosis with decrease in the bi-apical opacities and cystic lucencies as compared to before treatment

Follow-up chest X-ray showed bi-apical fibrosis with decreased bi-apical opacities and cystic lucencies (Figure 5), compared to pretreatment imaging. Monthly sputum studies remained positive for *M. szulgai*. A follow-up CT scan is planned six months after initiating therapy.

## Discussion

Out of more than 170 species of NTM identified, *M. szulgai* as a causative agent of pulmonary disease continues to be very rare, resulting in limitations in its clinical presentation, radiological findings, and treatment response.* Mycobacterium szulgai *is a slow-growing nontuberculous mycobacterium that has been increasingly recognized as a pathogen capable of causing human infections. While *M. szulgai* is commonly found in the environment, including water and soil, it is infrequently associated with disease in humans. However, when it is identified in clinical specimens, it often signifies true infection rather than colonization, necessitating prompt and adequate treatment to prevent disease progression.

*Mycobacterium szulgai* most commonly causes pulmonary infections resembling tuberculosis, with symptoms such as chronic cough, hemoptysis, fatigue, and weight loss. It has also been implicated in soft tissue infections, tenosynovitis, lymphadenitis, and, rarely, disseminated disease in immunocompromised individuals, including those with HIV, solid organ transplants, or malignancies. Reports of pleuritis and osteomyelitis caused by *M. szulgai *highlight its capability of causing localized, invasive disease [3].

*Mycobacterium szulgai* is a slow-growing NTM that exhibits intermediate virulence compared to other members of the *Mycobacterium* genus. It typically requires 2-4 weeks to form colonies [3]. While *M. szulgai* does not spread person-to-person like *M. tuberculosis*, it is considered one of the more pathogenic NTMs, capable of causing chronic pulmonary infections, skin and soft tissue infections, and disseminated disease in immunocompromised individuals [4]. Pulmonary infections caused by* M. szulgai* can mimic *M. tuberculosis*, often presenting with cavitary lung disease [5]. Its virulence is attributed to its ability to persist within macrophages, similar to *M. tuberculosis*, and its relative resistance to host immune clearance [6].

Isolation of *M. szulgai* from clinical cultures, particularly sterile sites or multiple respiratory specimens, is highly suggestive of pathogenicity rather than environmental contamination. According to the American Thoracic Society (ATS) and Infectious Diseases Society of America (IDSA) guidelines for NTM pulmonary disease, the diagnosis of true infection is supported by positive cultures from at least two separate sputum samples, positive culture from bronchial wash/lavage, or histopathologic evidence of granulomatous inflammation with positive NTM cultures from biopsy specimens [7]. Our case report met the condition of positive sputum samples; however, the patient declined bronchoscope and biopsy. Given its potential for significant morbidity, *M. szulgai* isolation must be taken seriously, and treatment should be initiated promptly once its pathogenicity is confirmed. Clinicians should be aware of such an organism as a potential cause of atypical respiratory infection.

*Mycobacterium​​​​​​​ szulgai* is generally susceptible to conventional first-line antimycobacterial agents, including concurrent treatment with clarithromycin, rifampin, and ethambutol, often used in combination therapy. For pulmonary infections, the IDSA and ATS recommend a three-drug regimen (clarithromycin or azithromycin, rifampin or rifabutin, and ethambutol) for at least 12 months after culture conversion. For localized infections, surgical debridement, when feasible, combined with prolonged antimycobacterial therapy, is often necessary. Lastly, for disseminated disease, treatment should be tailored based on susceptibility testing, with combination therapy to prevent resistance [8,9].

Persistent positive cultures in *Mycobacterium szulgai* pulmonary disease indicate ongoing bacterial presence despite treatment, suggesting either incomplete drug efficacy, patient non-adherence, drug resistance, or underlying host factors such as structural lung disease or immunosuppression [3]. According to the ATS/IDSA 2007 guidelines, treatment failure is defined as persistent culture positivity despite at least six months of guideline-based therapy, necessitating an evaluation of drug susceptibility, potential drug interactions, and patient adherence [3]. In cases of treatment failure, alternative regimens or salvage therapies may include the addition of an injectable aminoglycoside and the use of fluoroquinolones or clofazimine, or for last-line therapy, surgical resection can be considered [10].

## Conclusions

This case highlights the clinical significance of *Mycobacterium szulgai *as an emerging pathogen capable of causing severe infections, typically in individuals with underlying pulmonary conditions or weakened immune systems. While rare, the isolation of *M. szulgai *from clinical specimens should prompt careful evaluation to distinguish true infection from contamination, adhering to established diagnostic criteria. Early identification through culture and molecular techniques is crucial for the timely initiation of appropriate combination therapy, which is essential to prevent complications and ensure favorable outcomes. This case underscores the importance of considering *M. szulgai *in the differential diagnosis of chronic infections, raising awareness among clinicians about its pathogenic potential and management strategies.
